# Implementation Climate and Time Predict Intensity of Supervision Content Related to Evidence Based Treatment

**DOI:** 10.3389/fpubh.2018.00280

**Published:** 2018-10-04

**Authors:** Michael D. Pullmann, Leah Lucid, Julie P. Harrison, Prerna Martin, Esther Deblinger, Katherine S. Benjamin, Shannon Dorsey

**Affiliations:** ^1^Psychiatry and Behavioral Sciences, University of Washington School of Medicine, Seattle, WA, United States; ^2^Department of Psychology, University of Washington, Seattle, WA, United States; ^3^CARES Institute, Rowan University School of Osteopathic Medicine, Stratford, NJ, United States

**Keywords:** supervision, evidence-based treatment, implementation science, implementation strategies, trauma-focused cognitive behavioral therapy, measurement-based care

## Abstract

**Objective:** Children infrequently receive evidence-based treatments (EBTs) for mental health problems due to a science-to-practice implementation gap. Workplace-based clinical supervision, in which supervisors provide oversight, feedback, and training on clinical practice, may be a method to support EBT implementation. Our prior research suggests that the intensity of supervisory focus on EBT (i.e., thoroughness of coverage) during workplace-based supervision varies. This study explores predictors of supervisory EBT intensity.

**Methods:** Participants were twenty-eight supervisors and 70 clinician supervisees. They completed a baseline survey, and audio recorded supervision sessions over 1 year. Four hundred and thirty eight recordings were coded for supervision content. We chose to explore predictors of two EBT content elements due to their strong evidence for effectiveness and sufficient variance to permit testing. These included a treatment technique (“exposure”) and a method to structure treatment (“assessment”). We also explored predictors of non-EBT content (“other topics”). Mixed-effects models explored predictors at organizational/supervisor, clinician, and session levels.

**Results:** Positive implementation climate predicted greater intensity of EBT content coverage for assessment (coefficient = 0.82, *p* = 0.004) and exposure (coefficient = 0.87, *p* = 0.001). Intensity of exposure coverage was also predicted by more time spent discussing each case (coefficient = 0.04, *p* < 0.001). Predictors of greater non-EBT content coverage included longer duration of supervision sessions (coefficient = 0.05, *p* < 0.001) and lower levels of supervisor EBT knowledge (coefficient = −0.17, *p* = 0.013). No other supervisor- or clinician-level variables were significant predictors in the mixed effects models.

**Conclusion:** This was the first study to explore multi-level predictors of objectively coded workplace-based supervision content. Results suggest that organizations that expect, support and reward EBT are more likely to have greater intensity of EBT supervision coverage, which in turn may positively impact clinician EBT fidelity and client outcomes. There was evidence that supervisor knowledge of the EBT contributes to greater coverage, although robust supervisor and clinician factors that drive supervision are yet to be identified. Findings highlight the potential effectiveness of implementation strategies that simultaneously address organizational implementation climate and supervisor practices. More research is needed to identify mechanisms that support integration of EBT into supervision.

## Introduction

Many evidence-based treatments (EBTs) have been developed to address child and adolescent mental health needs (1). However, the potential promise of EBTs has not been realized due to the substantial challenge of implementing them in community mental health settings (2–4). Growing consensus in the literature indicates that EBTs are implemented at a slow pace in community settings, leading to critical gaps in the quality and effectiveness of mental health care (5–7). Experts have categorized over 70 implementation strategies (8), one of which is providing clinical supervision. Generally, clinical supervision is defined as an evaluative intervention wherein more senior clinicians provide oversight to more junior clinicians in order to ensure the quality of their services and provide ongoing clinical training (9). In the Exploration, Adoption/Preparation, Implementation, and Sustainment model of EBT implementation (EPIS) (10), fidelity monitoring and support—important aspects of EBT-focused clinical supervision—are specifically noted as inner setting factors affecting implementation. Without ongoing clinical supervision focused on the EBT, clinicians' fidelity can be low (11, 12), creating challenges for both the active implementation and sustainment phases of EBT implementation (10).

Clinical supervision focused on EBT delivery has been demonstrated to improve clinician EBT fidelity (13), knowledge, attitudes and skills (14). In relevant work from the expert consultation literature, in which EBT-specific supervision was provided by external EBT experts, a greater dose of EBT-focused supervision resulted in greater clinician skill in the EBT (15). Active learning strategies used in supervision (e.g., modeling) predicted community mental health clinicians' competent use of EBT strategies in the next therapy session (16). In an analog study that randomized psychology trainees into two groups (supervision as usual vs. supervision with active learning elements), only the active learning group had greater clinician knowledge, attitudes, and skill (14).

### Workplace-based supervision in community mental health organizations

One potentially sustainable way to increase clinician receipt of EBT-focused supervision in community settings with limited resources to support ongoing expert consultation is to identify existing organizational supports in which to embed EBT coverage. In a national survey, most community mental health organizations reported providing weekly workplace-based clinical supervision (17). Workplace-based supervision includes both clinical supervision as well as oversight for administrative issues, professional development, and emotional support, provided by internal staff employed within an organization (18). In a study by our research group examining workplace-based supervision within organizations participating in a state-funded EBT initiative, weekly occurrence of supervision was mostly upheld [75% reported weekly supervision for ~1 h (19)].

### Workplace-based supervision and EBT implementation

Very limited research has focused on workplace-based supervision and EBT implementation (20, 21). In one study examining discussion of evidence-based principles for behavior disorders, clinicians, and supervisors reported that EBT coverage was generally brief (20). In a study focused on Trauma-focused Cognitive Behavioral Therapy (TF-CBT) implementation (22), clinicians reported moderate coverage of TF-CBT elements in supervision (23). Schoenwald and colleagues (21) trained workplace-based supervisors in a manualized supervision model designed to support the implementation of Multisystemic Therapy (24). Supervisor adherence to treatment principles was related to increased treatment fidelity, and supervision structure was related to speed of change in client symptoms and functioning.

In a study on which the current investigation builds, Dorsey et al. (25) objectively coded the workplace-based supervision sessions of supervisors and clinicians participating in a state-funded TF-CBT initiative. TF-CBT is an evidence-based treatment for mental health sequelae subsequent to trauma exposure (26). It includes nine treatment elements (22): psychoeducation, parenting, relaxation, affect modulation, cognitive coping, trauma narrative, and processing trauma-related thoughts (imaginal exposure: facing up to memories of the traumatic event), *in vivo* mastery of trauma reminders (situational exposure: facing up to reminders in the environment), conjoint sessions, and enhancing safety. Many of these are used in other cognitive behavioral approaches to child and mental health disorders. Sixteen content areas, described in a measures table in Appendix A in Supplementary Material, were coded for occurrence and intensity of occurrence in the Dorsey et al. (25) study. These content areas included the nine TF-CBT elements as described in Table 1, some of which were collapsed for coding feasibility, as well as other content necessary for supervising TF-CBT (i.e., assessment, child's trauma history, use of art, play and books to engage children, treatment engagement), and clinician-level EBT techniques found to be infrequently used by clinicians in usual care (5) (e.g., assigning/reviewing client homework), and essential for effective delivery of TF-CBT [see Appendix A in Supplementary Material and (25) for more information on coding procedures]. There was substantial variation in content coverage, with some elements covered in more than half of the supervision sessions, and other important elements covered more rarely.

**Table 1:** Content elements of Trauma-Focused Cognitive Behavioral Therapy (TF-CBT).

**Content element**	**Goal**	**Description and Examples**
**PHASE 1: STABILIZATION AND SKILL BUILDING**		
Psychoeducation	Normalize parent and child's symptoms, provide information about responses to trauma, emphasize accurate thoughts about the event	Orienting the parent to the TF-CBT model by explaining the child's symptoms and the collaborative nature of treatment, and gives hope by describing the researched effectiveness of TF-CBT
Parenting skills	Improve parental functioning, which is related to child outcomes; structure and predictability enhances adaptive functioning for child and parent	Teaching and practicing functional analysis, praise, selective attention, time out, and contingency reinforcement
Relaxation skills	Reduce physiological symptomology related to anxiety or trauma	Teaching and practicing focused breathing, mindfulness, meditation, and progressive muscle relaxation
Affective modulation	Help children voice and handle their feelings more effectively with the goal of reducing avoidant strategies	Teaching and practicing feeling identification, thought interruption and positive interruption, positive self-talk, enhancing problem solving and social skills, managing difficult affective states
Cognitive coping	Explore thoughts and challenge maladaptive thoughts	Education about the cognitive triangle, and teaching to recognize types of inaccurate or unhelpful thoughts
**PHASE 2: TRAUMA NARRATION AND PROCESSING**		
Trauma narration and processing (exposure)^*^	Gradual imaginal exposure to the trauma and surrounding events, thoughts, and feelings to unlink trauma reminders to negative feelings	Talking and writing about the trauma gradually, but in detail, with the help of a therapist
**PHASE 3: CONSOLIDATION AND CLOSURE**		
*In vivo* mastery of trauma reminders (exposure)^*^	Exposure to objects and experiences to unlink trauma reminders to negative feelings. The only piece which is optional because most children do not overgeneralize fear to objectively non-threatening stimuli and so do not require *in vivo* exposure to combat functionally impairing avoidance	Gradually allow child to adjust to a feared situation that is objectively safe
Conjoint child-parent sessions	Encourages parents and children to practice skills together and to make the child more comfortable discussing the trauma with the parent	Parent should be carefully prepared to increase likelihood of positive interactions between parent and child in session
Enhancing future safety and development	Increase the likelihood of personal safety; especially important when there is potential for ongoing trauma	Developing a personal safety plan, teaching related skills: communicating feelings, attending to “gut feelings,” identifying safety cues, learning body ownership, recognizing secrets vs. surprises, and how to ask for help

* *In Dorsey et al. (25) and the current study these two elements were collapsed and coded as “exposure” to capture exposure content coverage in supervision*.

Potentially more important than whether an element is covered, is the *intensity* with which a supervisor covers EBT content. Following McLeod and Weisz's (27) operationalization, we define intensity as the frequency and thoroughness with which specific content elements are covered. As an example, the EBT of focus for this study, TF-CBT, includes two content elements focused on *exposure* (see Table 1, the trauma narrative imaginal exposure and *in vivo* exposure). These two exposure content elements were collapsed for coding of exposure content coverage in supervision sessions in the Dorsey et al. (25) study, on which this study builds [see Appendix A in Supplementary Material and Dorsey et al. (25) for more details about the coding procedures]. Exposure gradually reduces anxiety by having clients repeatedly face a feared stimulus, such as memories of a traumatic event. Intensity of exposure coverage during supervision would be determined by the extent of detail and time spent planning exposure content for an upcoming session or debriefing exposure coverage for a completed session. High intensity coverage of exposure may involve a detailed discussion of exposure use in the last session and planning for the next session (e.g., whether and how caregivers would be involved, ways to support the client during exposure, identifying strategies to manage client avoidance). Low-intensity coverage of exposure involves only a brief mention (e.g., “You should start the trauma narrative”). This low intensity coverage of EBT elements (e.g., a brief mention) is unlikely to provide sufficient fidelity monitoring or support. Similarly, *assessment* is a commonly used technique in TF-CBT that is discussed in supervision with varying levels of intensity. It is defined as the discussion of information about the client's psychosocial symptoms or behavior problems from standardized, formal assessment measures or functional analysis. Assessment is not one of the nine TF-CBT clinical content items but is necessary for delivering and supervising TF-CBT. High intensity coverage for assessment would involve the supervisor and clinician planning for assessment, reviewing assessment scores, and considering implications of scores for treatment. Low intensity would involve a brief mention of assessment without further discussion and would be unlikely to be related to any modifications in treatment planning or clinical approach.

### Study purpose and rationale

The current study extends Dorsey et al. (25) and seeks to identify clinician, supervisor, and organization characteristics that predict the intensity of coverage for two specific content elements important for workplace-based supervision of EBTs. By identifying the predictors of EBT-focused supervision content, this study can provide valuable information to optimize the effectiveness of clinical supervision as an implementation strategy. We chose to focus this study on the content elements of exposure and assessment for two primary reasons. First, there were statistical limitations that prohibited analyses predicting the variance of many other content elements. In the Dorsey et al. (25) study, both were among a small subgroup of content items that had sufficient overall variance in intensity of coverage and variance at the clinician and/or supervisor levels to permit the investigation of predictors. Second, exposure and assessment were selected from the subgroup because of their theoretical importance for TF-CBT implementation. We also focused on non-EBT related “other topics” content as an analytic counterpoint.

EBTs are generally comprised of multiple *clinical intervention elements*, but also include *structural elements* that support and organize technique delivery (28). *Exposure* was included in this study because it is a common and effective technique used in EBTs for child and adolescent anxiety disorders (29), is included in almost all EBTs for trauma treatment (19), and is one of the most active ingredients of TF-CBT (see Table 1). Exposure has very strong evidence for effectiveness (30); some studies even found exposure to be just as effective alone as when combined with other active components (31, 32). Despite the robust evidence supporting exposure, clinicians use it only rarely (27), possibly due to lack of comfort and training with this technique (33). *Assessment* is included in this study because it is a common and effective structural element that supports delivery of any EBT by assisting in planning for which EBT to use and if the client is having symptom improvement with receipt of the EBT. For flexible treatments, including TF-CBT, assessment also assists clinicians in deciding which clinical elements to deliver and when to deliver them. When used as part of routine outcome monitoring (e.g., repeated administration and review), it has been demonstrated to increase quality of care and improve outcomes (34, 35). Regular administration and review of client assessments helps focus clinicians on the needs of their clients and systematically identify progress or lack thereof (36). In TF-CBT, clinicians are expected to assess clients for trauma exposure and mental health symptoms before beginning treatment and to continue to assess clients' mental health symptoms throughout treatment to guide element ordering, dose (how many sessions allocated to any element), and to determine treatment response (i.e., is the client making progress?). The coverage of assessment in supervision could possibly facilitate thoughtful and timely treatment adjustments. Therefore, we chose to study assessment as a complement to exposure because assessment represents an evidence-based *structure* that supports treatment, rather than a specific clinical *element* like exposure.

In addition to examining predictors of two EBT content elements, we also wanted to examine predictors of *non-*EBT content coverage (i.e., *other topics*). *Other topics* was defined as discussion of issues unrelated to the child's traumatic experiences or TF-CBT practice components. This content may include the case background information, crisis, or case management, administrative work, and non-work related conversations. With limited time per case (25), the EBT focus of supervision could be “crowded out” by coverage of other topics that may be clinically relevant but do not directly support the clinician in the EBT. We included this variable as a negative control outcome (37) and a counterpoint to the other two EBT-focused dependent variables. Therefore, if a variable predicts intensity of *both* EBT and non-EBT content, we might conclude that it is simply a broad facilitator of intensity of supervision in general. However, if a predictor is positively related to EBT content intensity and *also* negatively related or unrelated to non-EBT content, it provides some empirical justification that the predictor may be a specific mechanism of intensity of EBT content coverage.

### Potential predictors of EBT content coverage in supervision

**Figure 1:**
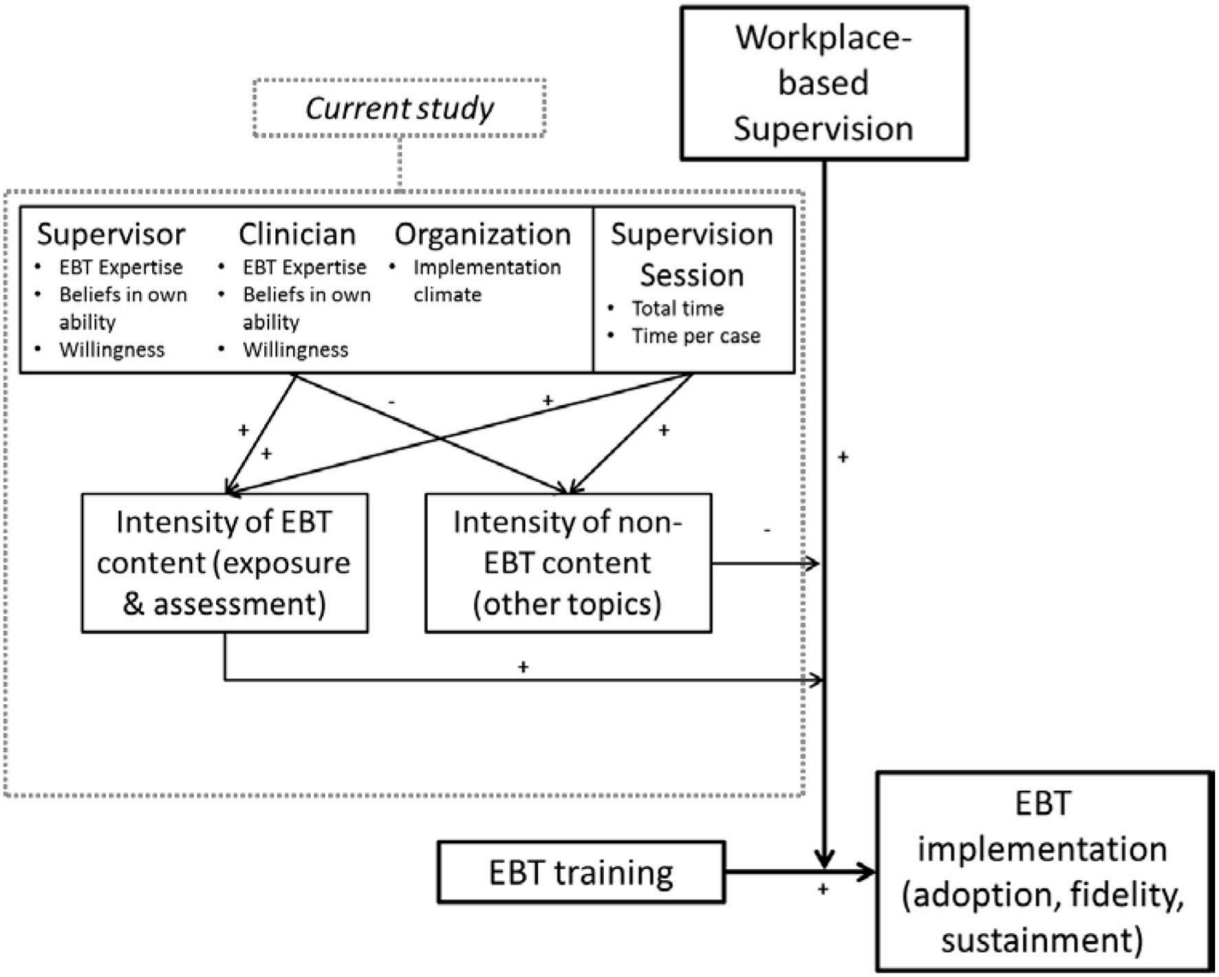
Hypothesized model of predictors of supervision content, within a broader theoretical model of supervision as an implementation strategy.

Because there is limited research on predictors of supervision content, we draw our hypothesized predictors from other supervision-focused research [e.g., (16)], theoretical models of supervision (38, 39), the expert consultation literature (40), and predictors of clinician EBT practice (41). Figure 1 displays our overall theoretical model and the placement of the current study within that model. Based on the studies described above, our overall theoretical model proposes that supervision acts as an implementation strategy that positively moderates the relationship between EBT training and EBT implementation (adoption, fidelity, and sustainment). The effectiveness of supervision as an implementation strategy is positively moderated by the intensity of EBT-related content delivered during supervision, and negatively moderated by the intensity of non-EBT content. In regards to the tested part of the model, we hypothesized that intensity of coverage for the two EBT-related supervision content areas, exposure and assessment, would be predicted by multiple characteristics of the organization, supervisor, clinician, and session, described in detail below.

#### Organizational factors

Implementation climate, defined as employees' shared perceptions of the degree to which innovation use is expected, supported, and rewarded (42), may be an important organizational-level predictor, given its role in theoretical models of organizational effectiveness (43, 44), though empirical work on implementation climate has been limited (43). If an organization expects, supports, and rewards EBT delivery, we believe supervisors and clinicians would be more motivated to address EBT-related content during supervision. A few cross-sectional studies have tested Klein & Sorra's model (42) and found support for the relation between implementation climate and implementation effectiveness [e.g., implementation of computer technology in schools (45) and physician enrollment of patients in clinical trials (46)]. In studies focused on workplace-based supervision, our research team has found an association between implementation climate and self-reported greater coverage of clinical versus administrative content (19) and intensity of TF-CBT-specific content (23). Therefore, we hypothesized that implementation climate will be positively associated with intensity of EBT content, and negatively associated with intensity of non-EBT content.

#### Supervisor factors

As supervision is an interpersonal interaction between the supervisor and the clinician supervisee, individual characteristics likely play a role in determining the nature of the interaction. Our hypotheses are informed by research findings that clinicians' training, experience, and skill have been associated with client outcomes (47, 48), that clinicians' years of experience has been associated with client satisfaction (49), and that clinicians' theoretical orientation has been associated with the use of EBT strategies in treatment (41). For EBT content to be covered during supervision, supervisors must have some *expertise* with the EBT (measured by their amount of training, whether they primarily use EBTs, and an objective EBT knowledge test), they must have a *belief in their own abilities* to cover EBT (measured by self-efficacy and self-rated skill), and they must have the *willingness* to cover EBT (measured by attitudes toward EBTs, CBT theoretical orientation, and comfort with providing supervision on specific EBT elements). Therefore, we hypothesized that these indicators would be positively associated with supervision intensity of EBT content and negatively associated with non-EBT content. We explored for the impact of other supervisor characteristics that although not specific to EBT, may play a role in supervision content coverage, including years of experience conducting therapy, percent of time providing supervision, and their own ongoing involvement in providing therapy.

#### Clinician factors

Clinician characteristics may also be associated with coverage intensity, perhaps directly through asking for or steering the supervision session in certain directions, or indirectly through supervisors' reactions to clinician characteristics. For instance, one study found that supervisors provided more professional development to clinicians whose clients demonstrated weaker improvements, possibly reflecting supervisors' perceptions of a need for improving clinical skill (21). Similar to supervisors, we felt that EBT content would be impacted by *clinicians'* expertise, belief in their own abilities to provide EBTs, and willingness to engage in the content. Therefore, we hypothesized that EBT training, objectively measured knowledge, self-efficacy, self-rated skill, attitudes toward EBTs, and CBT theoretical orientation would be positively associated with intensity of EBT content and negatively associated with non-EBT content.

#### Supervision session-specific factors

Intensity of supervision content is likely predicted by supervision session factors, specifically the overall time allocated to the supervision session and time allocated to any one client or case. Client caseloads in public mental health can be high. In the statewide initiative from which our sample was drawn, the average caseload was nearly 40 (19). Caseload size can limit EBT supervision time overall or time dedicated to any one case, which may in turn limit the possible intensity of supervision coverage of any single content area. In the objective coding study on which the current investigation builds (25), discussion of an EBT for any individual case averaged just under 12 min. We hypothesized that more time spent in supervision and more time per case would predict intensity of coverage for all three content elements (two EBT and one non-EBT).

## Methods

Data for the current study comes from a larger National Institute of Mental Health-funded study of workplace-based clinical supervision [see study protocol: (50)]. Participants were part of a state-funded EBT training initiative in public mental health in Washington State, which provides yearly in-person training and 6 months of expert consultation for TF-CBT [for more details on the training approach see (51)]. The current study uses objectively coded audio recordings of supervision collected during the “supervision as usual,” descriptive phase of the larger study and from baseline self-report surveys, prior to a subsequent randomized controlled trial (RCT) of two supervision approaches.

### Procedure

The overall procedure was that supervisors and clinicians provided consent and completed a measures battery at baseline. Over the course of the following year, supervisors audio recorded all of their supervision sessions and these were coded.

The study team first identified organizations that had participated in the state-funded EBT initiative and had at least one TF-CBT-trained supervisor still at the organization. Supervisors who agreed to participate then identified eligible clinicians from among their supervisees. The study team contacted these clinicians to invite their participation and obtain informed consent. Of those approached, 72% of the organizations, 76.7% of the supervisors, and 76% of the clinicians consented to participate.

### Data collection

Supervisor and clinician study participants completed one online self-report survey at the beginning of the study before participating in a 2-day TF-CBT booster and study procedures training. Both clinicians and supervisors received $30 for completing the surveys. Supervisors who participated in the study were asked to audio record the portions of their individual supervision sessions that pertained to participating clinicians' TF-CBT cases for one year (October, 2012–September, 2013). All audio recordings of these supervision sessions were sent to the study team. Supervisors did not record informal supervision sessions that occurred outside of regular supervision time or group supervision sessions. The audio recordings were saved on study-provided, password-protected tablet devices. The recordings were transferred to the study team using a cloud-based server compliant with the Health Insurance Portability and Accountability Act of 1996. Organizations that participated received $3,000 at the end of the RCT study.

The Washington State Institutional Review Board approved all study procedures.

### Participants

#### Supervisors

Table 2 presents demographic information for all participants. Participants for these analyses included 28 supervisors who submitted audio recordings, representing 17 public mental health organizations located in 23 separate offices. In order to meet study inclusion criteria, participants were required to have received TF-CBT-specific training as part of the EBT initiative, to be a current supervisor of a clinician in the study, to be currently employed at a public mental health organization, and to have no immediate plans to leave the organization. An additional 5 supervisors participated in this phase of the study but did not submit audio recordings and were therefore excluded from these analyses. As described elsewhere (25), there were few significant differences between supervisors who submitted or did not submit recordings, except that supervisors who submitted recordings were slightly older, more likely to endorse CBT as their primary theoretical orientation, and less likely to endorse family systems therapy or art/play therapy.

**Table 2:** Supervisor and clinician demographics.

**Variable**	**Supervisor (*****n*** = **28)**		**Clinician (*****n*** = **70)**	
	***n***	**%**	***n***	**%**
Female	18	64.3	61	87.1
**RACE/ETHNICITY**				
White/Caucasian	26	92.9	62	88.6
Hispanic or Latino	–	–	8	11.4
Asian	1	3.6	3	4.3
Native Hawaiian/Other	1	3.6	1	1.4
Black/African American	–	–	–	–
Other	–	–	2	2.9
**EDUCATION LEVEL**				
Bachelor's	–	–	5	7.1
Master's	26	92.9	62	88.6
Doctoral	2	7.1	3	4.3
**ACADEMIC DEGREE/BACKGROUND**				
Marriage/Family	5	17.9	8	11.4
Psychology	3	10.7	4	5.7
Social work	11	39.3	19	27.1
Counseling Psyc.	9	32.1	28	40.0
Other	–	–	11	15.7
**PRIMARY THEORETICAL ORIENTATION**				
CBT	21	75.0	45	64.3
Family systems	6	21.4	7	10.0
Solution-focused	1	3.6	3	4.3
Humanistic	–	–	4	5.7
Psychodynamic	–	–	7	10.0
Play therapy	–	–	3	4.3
Art therapy	–	–	1	1.4
Licensed	27	96.4	36	51.4
Mainly Uses EBT	21	75.0	51	72.9
	***M***	***SD***	***M***	***SD***
Age	44.4	10.4	38.0	11.5
Years providing therapy	14.1	7.6	7.0	6.2
Years at organization	10.4	6.4	4.7	4.1
Caseload size	12.6	12.1	30.1	12.6
Number of clinician supervisees	7.5	4.7	–	–
% Time on supervision	36.6	18.3	–	–
% Time on clinical work	26.9	20.5	–	–
Number of different types of TF-CBT training	5.0	1.8	3.9	2.0

#### Clinicians

Participants included 70 clinicians who were recorded in supervision sessions. Eligibility criteria for clinicians to participate in the study included: trained in TF-CBT through the statewide initiative, currently provide TF-CBT to children and adolescents, supervised by a supervisor involved in the study, employed at least 80% full-time equivalent or more, no immediate plans to leave the organization, and provided therapy in English (to enable coding of TF-CBT fidelity for other analyses). An additional 15 clinicians participated in this phase of the study, but audio recordings of their supervision sessions were not submitted, and they were therefore excluded from the study. As reported elsewhere (25), clinicians who were recorded and not recorded differed only on a few variables: clinicians who were recorded had provided psychotherapy for longer and were less likely to have a degree in Marriage and Family Therapy.

### Measures

Below, we describe the measures used in this study. For additional information, see the measures table in Appendix A in Supplementary Material.

#### Implementation climate

Supervisors and clinicians completed the six-item Evidence-Based Organizational Checklist to assess the level to which their organizations expect, support, and reward EBT. All participant scores within each organization were aggregated to create an organizational implementation climate score. The content in this measure is similar to that of another implementation climate measure that was not available when the study began (52). Items are rated on a 4-point Likert scale (1, never; 2, occasionally; 3, most of the time; 4, ongoing/routine). Example items from this measure include, “Executive leadership (e.g., administrators, directors) explicitly and repeatedly express support for and promote use of EBT,” and “Clinicians are provided with EBT training opportunities and ready access to EBT materials (manuals, handouts, equipment).” Previous studies have verified the unidimensionality and internal reliability of measure scores [see (51)]; the current study replicated good internal reliability (Cronbach's α = 0.86). Higher scores indicate a more supportive EBT implementation climate. Construct validity of the measure is supported by a significantly high office-level Intraclass Correlation ICC_(1, 1)_ of 0.41. We use the ICC here to indicate “validity” rather than “reliability” because the clustering of implementation climate ratings by members of the same office indicates that climate is a shared perception at the office level (53, 54). Due to the small number of supervisors per office and challenges with a four-level model, we included implementation climate in analyses at the supervisor level (e.g., two supervisors in the same office would have the same climate score).

#### Participant characteristics

Supervisors and clinicians were asked to provide information on their age, sex, ethnicity, race, number of years they had conducted therapy, and whether they felt they mainly used EBTs in their work. Participants indicated the total number of different types of training experiences they had with TF-CBT out of 12 possible options (e.g., “completed a 2-day in-person training,” “read the 2006 TF-CBT book”); experiences were summed. Participants endorsed their primary theoretical orientation from a list of 10 possible options. Supervisors provided an estimate of the percentage of time they spent providing supervision, whether they still actively performed clinical work, and chose the TF-CBT element that they felt was most difficult to supervise, which we transformed into a variable indicating whether or not they chose exposure as the most difficult element to supervise.

#### TF-CBT self-efficacy

Supervisor and clinician self-efficacy in TF-CBT was assessed using an 11-item index adapted from two previous measures (55, 56). Participants rated their level of competence implementing TF-CBT on a 5-point Likert scale (0, not at all; 1, a little bit; 2, somewhat; 3, very much; 4, exceptionally) using items such as “Completing trauma narratives with children,” and “Analyzing complex clinical situations from a TF-CBT perspective.” An exploratory factor analysis using maximum likelihood extraction in the current sample justified retaining a single factor accounting for 56% of the variance; Cronbach's alpha was 0.92.

#### Declarative knowledge and skill with TF-CBT and exposure

The Skill in Implementing Components: Trauma and PTSD scale was used with supervisors and clinicians to obtain the self-reported understanding of and skill in the major components of CBT for cases with trauma and PTSD (51). It includes 11 items rated on a 6-point scale ranging from 0 (do not use) to 5 (advanced), and asks participants to rate their understanding and skill of elements such as “Psychoeducation” and “Cognitive Coping.” In psychometric testing using an earlier version of this measure that asked about other elements in addition to trauma and PTSD, the trauma and PTSD scale emerged as a clear factor (51). Data from the current study had very high internal consistency (Cronbach's α = 0.91). We used the total mean score as well as a mean of two items, “*in vivo* Exposure” and “Trauma narrative.”

#### TF-CBT knowledge

Supervisors and clinicians completed a 13-item multiple choice test of TF-CBT knowledge that combines items from the Denver Post Health Survey (57) with items added by our team, and includes content similar to the knowledge test used for the clinician TF-CBT certification program (https://tfcbt.org). Participants provided multiple choice or true/false response ratings to items such as “When teaching cognitive coping, wait to challenge distorted/unhelpful cognitions related to trauma.” The measure has been found to have a good response range for item difficulty and item discrimination, and has demonstrated convergent validity with number of trainings and TF-CBT self-efficacy (19).

#### EBT attitudes

Supervisors and clinicians completed the Modified Practice Attitudes Scale (MPAS) to assess attitudes toward EBTs (58). The current study used a five-item version of the MPAS with acceptable internal consistency and good validity (59). Participants indicated their agreement with statements such as “Clinical experience and judgment are more important than using evidence-based treatments,” using a 4-point scale ranging from 0 (not at all) to 4 (to a very great extent). The current study found acceptable internal consistency (Cronbach's α = 0.78).

#### Supervision session time

During coding of audio recordings (described below), coders determined the length of the supervision session (in minutes) and number of cases discussed. Average minutes per case was calculated by dividing the total session time by the number of cases.

#### Supervision content

The dependent variables used in this study, i.e., intensity of supervision content areas, were obtained using the Supervision Process Observational Coding System (SPOCS), which was adapted from the Therapeutic Process Observational Coding System for Child Psychotherapy—Strategies scale [TPOCS-S; (27, 60)]. The TPOCS-S categorizes psychotherapy treatment intervention elements using direct observation. Similarly, the SPOCS categorizes supervision elements, applying Garland et al.'s (5) adaptation of the TPOCS-S by stratifying codes into content and technique domains. For the current study, we focused only on the content domain.

There are 16 content areas in the SPOCS, described in detail elsewhere (25) and in Appendix A in Supplementary Material. We examined three content items for the purposes of the current paper: exposure [which combines two exposure elements: (a) trauma narration and processing and (b) *in vivo* mastery of trauma reminders, see Table 1], assessment, and other topics (including crisis or case management). As reported in Dorsey et al. (25) trained coders rated content in 5-min intervals and then considered ratings across intervals to generate an overall intensity score for each individual content item. These three content items had normally distributed intensity scores, with ratings from 0–6, (0 = not present, 1–2 = low intensity, 3–4 medium, 5–6 = high). For instance, low intensity ratings for assessment reflected brief mentions of the content (e.g., “Don't forget to do the weekly assessment”). High intensity ratings for assessment reflected more in-depth discussion, such as planning for assessment in an upcoming session (rationale, strategies to remove barriers), in treatment generally, and/or review of assessment results (e.g., scores, clinical significance, change over time) and implications for the treatment plan (such as whether assessment scores indicate that a specific component is warranted). As the SPOCS is newly developed and there is no existing measure with which to compare, we lack complete psychometrics on this measure. However, as described below, the coding team achieved very high interrater reliability, which suggests that the SPOCS identifies distinct and observable components.

### Coder training and session sampling

#### Coder training

The details of SPOCS coder training are described elsewhere (25). Coders were six post-baccalaureate research assistants. Coders attended an initial training, which included content review, group coding, and detailed coding manual review and discussion. They then coded 10 training files independently to ensure satisfactory interrater reliability across group members and with the last author. Official coding for the study began once each coder's ratings reached an established criterion: interrater reliability using two-way random single measure intraclass correlation coefficients [ICC_(2,1)_ ≥ 0.80; (61)]. For individual content/technique items for which an ICC_(2,1)_ ≤ 0.60, coders were required to engage in additional practice and review. Coders were required to re-read the coding manual monthly, discuss, and reference the manual when questions or confusion arose, and attend recurring booster trainings to prevent drift. Coders were randomly assigned supervision files. Possible rater drift was monitored through masked coders double-coding sessions at regular intervals; ICCs remained strong throughout and no coder fell below an ICC_(2,1)_ of 0.80.

#### Session sampling procedures

In total, we received 638 supervision recordings. Per supervisor, up to 23 individual supervision sessions were coded (when available), resulting in 438 coded recordings. When a supervisor submitted 23 or fewer files, the study team coded all submitted files. When a supervisor submitted over 23 files, 23 files were randomly selected using a form of stratified random sampling in which selected recordings were distributed across time and participating clinicians.

#### Interrater reliability

To test interrater reliability, 105 (23.9%) of the 438 sampled session recordings were coded by multiple coders. The overall group average ICC assessing reliability was ICC_(2,6)_ = 0.87, representing excellent reliability (61). Coders had excellent individual ICCs of 0.84 or higher. At the item level, ICC_(2,1)_ statistics ranged from good to excellent, Exposure = 0.92, Assessment = 0.76, Other topics = 0.85.

### Analyses

Analyses were conducted in SPSS 19. Means, standard deviations, and percentages were calculated for participant descriptive information and content items. Using null models with no predictors, three separate 3-level mixed effects models with random intercepts at the supervisor and clinician level (supervision session nested within clinician nested within supervisor) were used to compute intraclass correlations (ICCs), which are the proportion of variance for each dependent variable attributable to each level. Although 4-level models that include nesting within organization would be more appropriate, several organizations had only a single supervisor participating in the study, and therefore, clustering estimates for these models failed to converge. Restricted Maximum Likelihood (REML) estimation and an unstructured covariance matrix were used to obtain final parameter estimates.

Model building for hypothesis testing followed standard protocol (62). For each unique independent-dependent variable combination, a separate model was computed, which tested the unique bivariate relationship between each independent and dependent variable, similar to the standard practice of computing a correlation table prior to ordinary least squares regression modeling. Based on these analyses, we built models beginning with level 1. All predictor variables were entered as grand mean centered to aid interpretation of the intercept—using this approach, the intercept represents the estimated mean score of the dependent variable, rather than the estimated score if all predictors were zero. Level-1 and level-2 predictors were entered in bivariate analyses as fixed effects and then as random effects. In all models below, no randomly varying slopes were significant, and allowing the effects of these level-1 predictors to vary did not improve model fit, or models failed to converge; thus, all level-1 and level-2 slopes in all models were fixed. We removed or retained parameters based on model fit statistics, assessed using significance of−2 log likelihood deviance and magnitude of Bayesian Information Criteria (BIC) deviance, with values for the BIC above 2 considered positive evidence of model superiority, and values above 10 indicating strong evidence (63). After a level-1 model was built, each level-2 predictor that had been significant at *p* < 0.05 during the bivariate testing described above was added as a fixed effect in a stepwise fashion to assess model fit. When two or more variables were individually significant but non-significant when jointly entered, model fit statistics were used to determine the best fitting parsimonious model.

## Results

### Exposure

#### Contextual information; Dorsey et al. (25) analyses

As originally reported in the study on which our investigation builds (25), exposure was frequently covered, in that it was mentioned in 82% of the coded supervision sessions. The intensity of exposure coverage varied, however. In 17% of sessions it was not discussed, in 24% of the sessions it was discussed with low intensity, in 41% with medium intensity, and in 17% with high intensity (*M* intensity across sessions = 2.64, *SD* = 1.75). A null (no predictor) model predicting intensity of exposure coverage indicated that 16% of the variance in exposure coverage was at the supervisor level and 19% at the clinician level, with the remaining 65% at the individual supervision-session level. Therefore, intensity of supervision time spent on exposure appeared to be attributable to factors at both the supervisor and clinician levels.

#### Current analyses

Item range, means, standard deviations for each predictor variable are depicted in Table 3. Bivariate models for each potential predictor of intensity of exposure coverage at the organization, supervisor, clinician, and supervision-session level resulted in few significant associations (see Table 3). Longer TF-CBT supervision sessions, more supervision time per case, supervising a clinician with a cognitive behavioral theoretical orientation, and a more positive organizational implementation climate were associated with greater intensity coverage of exposure in supervision. Supervisors' belief that exposure or the trauma narrative was the most difficult element to supervise was associated with lower intensity of exposure coverage.

**Table 3:** Predictor descriptives and mixed linear model coefficients showing bivariate associations among supervision content and characteristics of the supervisor, clinician, and supervision session.

	**Range**	**Mean (*SD*)**	**Exposure^a^**	**Assessment^a^**	**Other topics^a^**
**ORGANIZATIONAL CHARACTERISTICS**					
Implementation climate	1.8–3.8	3.1 (0.53)	1.039^*^	0.818^*^	−0.654
**SUPERVISOR CHARACTERISTICS**					
**Expertise**					
# of TF-CBT trainings	1–10	4.7 (2.0)	0.124	0.153	−0.260^*^
Primarily uses EBT	0–1	0.75 (0.44)	0.677	0.797^*^	−0.639
TF-CBT knowledge test	6–13	10.1 (1.7)	0.140	0.100	−0.302^*^
**Beliefs in own ability**					
TF-CBT efficacy	2.5–4.6	3.6 (0.49)	0.804	0.446	−0.273
Declarative TF-CBT knowledge/skill	2–5	3.8 (0.69)	−0.038	0.246	−0.204
Declarative exposure knowledge/skill	2–5	3.6 (0.93)	0.061	NA	NA
Believes exposure is most difficult to supervise	0–1	0.52 (0.50)	−0.505^*^	NA	NA
**Willingness**					
EBT attitudes	2.6–5	4.2 (0.50)	0.263	0.416	0.331
**Theoretical orientation**					
Cognitive behavioral	0–1	0.68 (0.47)	0.015	0.933^*^	−0.189
Family systems	0–1	0.18 (0.39)	−0.256	−0.859^*^	−0.084
**Other practice characteristics**					
Number of years conducting therapy	3–37	12.1 (6.8)	0.025	−0.010	−0.042
% time providing supervision	5–90	37.5 (20.1)	0.005	0.001	0.005
Currently provides clinical services	0–1	0.86 (0.35)	0.570	0.533	0.570
**CLINICIAN CHARACTERISTICS**					
**Expertise**					
Number of TF-CBT trainings	1–9	3.3 (1.76)	−0.013	−0.044	0.067
Number of years conducting therapy	1–30	4.8 (5.01)	−0.007	0.007	−0.005
Primarily uses EBT	0–1	0.77 (0.42)	0.391	0.120	0.264
TF-CBT knowledge test	3–13	9.0 (1.90)	0.021	−0.071	−0.044
**Beliefs in own ability**					
TF-CBT efficacy	1–5	3.1 (0.70)	0.051	−0.076	0.062
Declarative TF-CBT knowledge/skill	1–5	3.1 (0.80)	0.043	0.056	0.196
Declarative exposure knowledge/skill	1–5	3.0 (1.05)	0.087	NA	NA
**Willingness**					
EBT attitudes	2.4–5	3.9 (0.49)	0.179	0.243	0.086
**Theoretical orientation**					
Art therapy	0–1	0.01 (0.11)	−0.395	−0.140	−0.676
Cognitive behavioral	0–1	0.65 (0.48)	0.745^*^	0.388	−0.312
Family systems	0–1	0.09 (0.29)	−0.272	0.562	−0.049
Humanistic	0–1	0.06 (0.23)	0.435	−0.676	0.080
Play therapy	0–1	0.03 (0.18)	−0.458	−0.233	0.017
Psychodynamic	0–1	0.08 (0.27)	−0.904	−0.313	0.716
**SUPERVISION SESSION CHARACTERISTICS**					
Supervision session duration in minutes	1–72	21.6 (15.0)	0.025^*^	0.009	0.051^*^
Minutes per case	1–51	12.2 (8.6)	0.034^*^	0.006	0.025^*^

* p < 0.05

a *Each supervision content area refers to the intensity with which the clinical content was discussed during supervision sessions. Exposure is defined as discussions of a technique to gradually reduce fears and anxiety by subjecting the client to a feared stimulus, such as memories of a traumatic event. Assessment is defined as discussions of information about the child's psychiatric symptoms or behavior problems from standardized, formal assessment measures and functional analysis. Other topics is defined as discussions of issues unrelated to the child's traumatic experiences or not directly related to TF-CBT components*.

The final model predicting exposure is depicted in Table 4. Average estimated exposure intensity was 2.7. Time spent per case was significantly and positively associated with exposure intensity, with each additional minute of time related to a 0.04 increase in intensity. Implementation climate was also significantly and positively associated, with each additional one-point increase in implementation climate associated with a 0.87 increase in exposure intensity. Therapists with a CBT orientation had an average exposure intensity score 0.52 points higher than those with another orientation, as indicated by improved model fit (Δ-2LL_(1)_ = 37.6, *p* < 0.001) and strong evidence of model superiority (ΔBIC_(1)_ = 37.7) when compared to a model without this variable. However, the individual variable parameter did not meet statistical significance (*p* = 0.112), so cautious interpretation is warranted. The final model accounted for 12.5% of the overall variance. This included 3.5% of the variance at the individual supervision-session level and 66.5% of the variance at the supervisor level. Variance at the clinician level slightly increased from the null to the final model.

**Table 4:** Mixed linear model predicting intensity of exposure coverage in workplace-based supervision.

	**Coefficient**	***SE***	***t***	***p***	
Intercept	2.66	0.15	18.15	< 0.001	
Supervision session minutes per case	0.04	0.01	3.99	< 0.001	
Therapist: CBT orientation	0.52	0.32	1.61	0.112	
Implementation climate	0.87	0.32	2.73	0.001	
**Variance components**	**Estimate**	***SE***	***Z***	***p***	**% variance accounted for**
Residual (session duration)	1.97	0.15	13.41	< 0.001	3.5%
Supervisor	0.17	0.22	0.75	0.456	66.5%
Clinician	0.60	0.25	2.41	0.016	−1.7%
Overall	2.73				12.5%

### Assessment

As reported elsewhere (25), compared to exposure, assessment was more rarely discussed, and included in only 55% of the coded supervision sessions. The intensity of assessment coverage varied, with only a few sessions addressing it with high intensity (in 45% of the sessions it was not discussed, in 32% it was discussed with low intensity, in 18% with medium intensity, and in 5% with high intensity; *M* intensity across sessions = 1.30, *SD* = 1.52). A null model indicated that 23% of the variance clustered at the supervisor level, only 2% clustered at the clinician level, and the remaining 75% of the variance was at the individual supervision-session level, implying that clinician-level factors likely do not account for any significant amount of assessment coverage during supervision.

#### Current analyses

Consistent with this, bivariate models found that assessment was significantly associated only with supervisor-level variables. Higher assessment intensity scores were associated with supervisors who reported that they primarily used EBTs, more positive implementation climate, and supervisors who reported having a CBT orientation. Lower assessment intensity was associated with supervisors who reported having a family systems theoretical orientation.

The final model for assessment is depicted in Table 5. Average estimated assessment coverage intensity was 1.3, and it was predicted only by implementation climate (each one-point increase in implementation climate was related to a 0.64 increase of intensity). The model accounted for 7.3% of the overall variance, mostly due to accounting for 32.8% of the variance specifically at the supervisor level; clinician variance slightly increased.

**Table 5:** Mixed linear model predicting intensity of assessment coverage of workplace-based supervision.

	**Coefficient**	***SE***	***t***	***p***	
Intercept	1.31	0.14	9.67	< 0.001	
Implementation climate	0.82	0.26	3.16	0.004	
**Variance components**	**Estimate**	***SE***	***Z***	***p***	**% variance accounted for**
Residual (session duration)	1.76	0.13	13.64	< 0.001	0%
Supervisor	0.36	0.13	2.38	0.017	32.8%
Clinician	0.05	0.08	0.56	0.575	−6.0%
Overall	2.16				7.3%

### Other topics

#### Contextual information; Dorsey et al. (25) analyses

As reported elsewhere (25), other topics was the content item delivered in almost every coded supervision session (96%). It was covered with the greatest mean level intensity (3.46, *SD* = 1.47), although intensity did vary across coded sessions (4% not discussed, 19% discussed with low intensity, 50% discussed with medium intensity, 27% discussed with high intensity). A null model found that 34% of the variance in intensity of other topics was at the supervisor level, 8% was at the clinician level, and the remaining 58% was at the individual supervision session level.

The “other topics” that were discussed consisted of case background information (45%), administrative work (15%), case management (10%), child symptoms and behavior problems (10%), non-trauma focused treatment elements (10%), non-work related conversations (8%), and crisis management (2%). Bivariate models found that intensity of supervisory time spent on other topics was predicted by duration of the session, minutes per case, lower supervisor scores on the TF-CBT knowledge test, and less supervisor-reported training in TF-CBT.

#### Current analyses

The final model for other topics is depicted in Table 6, and estimated the average intensity of supervisory time spent on other topics at 3.5. Other topics was predicted by the duration of the supervision session (each additional minute was associated with a 0.05 increase of other topic intensity), and supervisors with lower scores on the TF-CBT knowledge test spent more time on other topics (each additional point on the knowledge test was associated with a 0.17 *decrease* in other topic intensity). Overall, the model accounted for 39.2% of the variance in other topics intensity. This included 14% of the variance at the supervision session level and 92.6% of the variance at the supervisor level.

**Table 6:** Mixed linear model predicting intensity of “other topics” coverage in workplace-based supervision.

	**Coefficient**	***SE***	***t***	***p***	
Intercept	3.46	0.09	39.16	< 0.001	
Supervision Session Duration	0.05	0.01	11.22	< 0.001	
Supervisor TF-CBT Knowledge	−0.17	0.06	−2.72	0.013	
**Variance components**	**Estimate**	***SE***	***Z***	***p***	**% variance accounted for**
Residual (session duration)	1.09	0.08	13.57	< 0.001	14.0%
Supervisor	0.05	0.08	0.72	0.473	92.6%
Clinician	0.18	0.09	20.59	0.039	−1.0%
Overall	1.32				39.2%

## Discussion

Workplace-based supervision might be an effective dissemination and implementation strategy to increase the adoption, fidelity, and sustainment of EBTs. This study used an innovative method of coding supervision elements to explore the predictors of EBT content delivery during workplace-based supervision. To our knowledge, this is the first study to use objectively coded data from workplace-based supervision of EBT to explore predictors of intensity of coverage of EBT content. We found some support for multi-level predictors at the organization and supervisor levels, and as hypothesized, time played an important role. However, surprisingly few supervisor-level and no clinician-level variables predicted intensity of coverage for either of the two EBT content areas (i.e., exposure and assessment) or for other topics in multivariate models, and a large amount of variance remained unexplained for the two EBT content areas.

Implementation climate predicted intensity of coverage for both exposure and assessment. Overall, results suggest that a climate that supports, expects, and rewards EBT use may be one of the most important factors for improving the degree to which supervisors cover EBT in their supervision sessions. This finding is in line with two other supervision content-focused studies in which implementation climate was a significant predictor of clinician-reported supervision time spent on case conceptualization and interventions (19) and intensity of TF-CBT content (23). The present study is a *constructive replication* (64) of this prior work in that it analyzes objectively coded data instead of self-report, providing stability for these findings. Of the variables we explored, implementation climate was the strongest predictor. After controlling for other significant covariates, each one-point increase in climate was associated with a nearly one-point increase in intensity of exposure and assessment coverage, on a 6-point scale. These findings indicate that supervisors in organizations with more positive implementation climates may be more likely to provide the fidelity monitoring and support necessary as an inner context support during the latter two EPIS phases, active implementation and sustainment (10). The findings highlight the importance of creating an environment within which supervisors feel supported to carve out supervision time to cover EBT in greater intensity and feel that this is expected and rewarded in their organizations, despite competing demands on limited supervision time in the context of clinicians' high caseloads.

In light of our findings, it is important to consider that the association between supervision and implementation climate is likely bidirectional; supervisors both create and are shaped by implementation climate. Other studies on clinical supervision have raised similar questions surrounding this bidirectionality. For example, an observational study which adapted a supervision model from Multisystemic Therapy to implement social-emotional interventions in schools (65) raised questions about “the extent to which the scope of clinical supervision, and responsibility of the clinical supervisor, extends to the proactive cultivation and maintenance of organization-intervention fit …” (p. 55). Relatedly, Birken and colleagues proposed that “middle managers,” defined as employees who supervise frontline staff and are themselves supervised by top organizational leaders, play several key roles hypothesized to positively impact implementation climate and implementation effectiveness (66). As middle managers, supervisors go beyond providing clinical oversight, and regularly support EBT implementation at their organizations. Studies testing the impact of supervisor-level interventions on implementation climate and effectiveness are currently underway [e.g., (67)], and more studies are needed to further unpack the relationship between supervision and implementation climate.

Our hypotheses that time would be positively related to coverage intensity were supported for exposure and other topics but, interestingly, not for assessment. Among other determinants of practice, the lack of time is frequently endorsed by clinicians and other healthcare providers as a substantial barrier to EBT implementation [e.g., (68–71)]. The role that time plays appears to be complex. For exposure, *more time per case* was a stronger predictor than time allotted to the EBT supervision overall. In the objective coding study on which the current study builds, the average supervision time dedicated to a specific case was just over 12 min (25). Assuming a linear relationship, supervision time for any individual case would need to be doubled, from 12 to 24 min, to obtain a 1-point increase in intensity of exposure coverage in supervision. For the intensity of coverage of other topics, *time allotted to the EBT supervision overall* was a stronger predictor than time per case. For every minute increase in session duration, we saw a small (0.05) increase in the intensity of other topics. Most of the content areas that comprise other topics were related to the case being supervised, and primarily included discussion of case background information (about half of the content). However, off-topic or administrative content made up nearly a quarter of the other topics' content. Of all 16 areas coded, the variance attributable at the supervisor-level was the highest for other topics [34%; (25)]; therefore, more time may permit supervisors to focus on other topics beyond the EBT with greater intensity. Questions remain regarding this relationship: it could be that other topics conversations in supervision lead to lengthier supervision sessions, or it could be that shorter supervision time has the effect of enabling greater efficiency and strategic use of time to focus more on EBT.

Only one supervisor characteristic was significantly associated with any of our dependent variables after controlling for other variables. Supervisors with less knowledge of the specific EBT (TF-CBT) covered other topics with greater intensity. This finding makes logical sense, as EBT expertise would seem to be a requirement for greater intensity of coverage. Other significant bivariate associations, although not supported in the multivariate models, also suggest that supervisor-specific expertise and experience may play a role in intensity of content coverage (e.g., lower supervisor comfort supervising exposure was associated with *less* intense exposure coverage; supervisors primarily using EBTs and having a CBT theoretical orientation were associated with *greater* intensity of assessment coverage; supervisors with more TF-CBT training were associated with *less* intense other topics coverage). However, our hypotheses about multiple other variables being associated with content had no support even at the bivariate level (e.g., TF-CBT-specific self-efficacy, declarative knowledge and skill with TF-CBT, years of experience).

Similarly, the lack of empirical support for clinician-level predictors for any of the three content elements was unexpected. Neither of the variables capturing clinician expertise with the EBT was significantly associated with coverage intensity for any of the three supervision content elements. This was particularly surprising for exposure, which was unique among the three content areas in that it clustered with more than 10% of the variance at *both* the clinician and supervisor levels. Even in bivariate analyses, there was only one significant clinician-level predictor, and it was associated with willingness to address EBT: clinicians' self-report of a CBT theoretical orientation was related to more intense exposure coverage. This suggests that while the makeup of supervision may be driven in some small part by contributions of the clinician and the supervisor, the tailoring of supervision content to the needs of the clinician (e.g., based on skill or experience) may occur less frequently than our team predicted.

While our model for other topics was strongly predictive, with supervisor knowledge and supervision session length explaining nearly 40% of the overall variance, models for exposure and assessment did not explain much variability (13 and 7%, respectively). Based on these ICCs, it appears that much of the variance in delivery of these two EBT content elements occurs at the supervision-session level. There are three possible reasons for variability at this level: general random error, measurement error associated with coding reliability, and true session-level differences; unfortunately, these sources of variability are not statistically separable. The very high coder interrater reliability indicates that measurement error is not likely to be a major source of variance. Therefore, session-level sources of variability likely arise from multiple variables for which we do not have data, including the specific session-level needs of the clients, the timeline of treatment (e.g., assessment may be more likely to be discussed early in the treatment process), and the moods and cognitive loads of clinicians and supervisors during any specific supervision session. These variables can act as statistical noise, creating challenges for detecting predictors.

This study has a number of strengths. Findings are backed by strong internal validity from the use of our objective coding measure for supervision, and they replicate findings from analyses using self-reported data. Supervision session data was obtained from actual workplace-based supervision sessions from participants in a statewide EBT implementation initiative, providing generalizability to other EBT implementation efforts that attempt to leverage workplace-based supervision of EBTs. However, there were some limitations. Many of the content elements we coded (e.g., coverage of cognitive processing, clinician modeling, and role-play during treatment sessions) were not analyzed due to limited variability (i.e., rare occurrence; occurred with low intensity). Although the sample size for number of recordings was high (438 recordings), due to the nested nature of the data, the sample size for supervisors and clinicians (28 and 70, respectively) limited our power to detect effects at these levels. Also, as described previously, many variables that explain session-level variance were unmeasured (e.g., client needs/progress, clinician/supervisor temporal mood). The data are correlational and causal direction cannot be demonstrated. Additionally, to protect sensitive client information, supervisors were asked to only record the portion of individual supervision that pertained to TF-CBT cases. Similarly, we did not sample or code informal “drop-in” or group-based supervision, all of which may also contribute to the supervision of any one case.

Considering practical implications for workplace-based clinical supervision as a support for EBT implementation, our findings suggest that spending more time on supervision may not be the most efficient method to heighten the EBT focus of supervision. Time was not a significant predictor for assessment, and to increase intensity of coverage for exposure, a substantial and likely infeasible amount of time would need to be added. Simply increasing the amount of time for supervision might also result in supervision that is less focused on EBT content (i.e., more time focused on other topics). In contrast, having a more positive implementation climate had a strong effect. This suggests that efforts to improve the degree to which individuals perceive that their organization supports, expects, and rewards EBT use may positively impact the EBT focus of supervision and in turn support higher fidelity EBT delivery by clinicians. However, the field is only beginning to examine practical and effective methods for enhancing implementation climate. Future research on impacting supervision structure could explore the feasibility of improving implementation climate and the nature and direction of the relationship between organizational climate and supervisor behaviors. Meanwhile, additional research could be conducted to better identify the supervisor, clinician, and client-level variables that explain EBT and other content coverage in supervision.

## Ethics statement

This study was carried out in accordance with the recommendations of the Washington State Institutional Review Board, within the State of Washington Department of Social and Health Services. The protocol was approved by the Washington State Institutional Review Board. All participants gave written informed consent in accordance with the Declaration of Helsinki.

## Author contributions

MP was responsible for conceptualizing, analyzing, and writing this manuscript. LL, JH, PM, and KB were responsible for conducting project activities and writing the manuscript. LL and KB also coded supervision audio files. SD and ED were responsible for developing the supervision coding manual, directing project activities, conceptualizing, and writing.

### Conflict of interest statement

SD and ED have received honorariums for providing TF-CBT training. The remaining authors declare that the research was conducted in the absence of any commercial or financial relationships that could be construed as a potential conflict of interest.
