# Supplementary material for: Implementation Climate and Time Predict Intensity of Supervision Content Related to Evidence Based Treatment
Source: Front Public Health. 2018 Oct 4;6:280. doi: 10.3389/fpubh.2018.00280 (PMC6180155; doi:10.3389/fpubh.2018.00280)
Supplement: Supplementary file 1 [file Data_Sheet_1.docx]

Appendix A.

*Table of measures*

| **Construct/**  **Measure** | **Description** | **# Items / Scale** | **Psychometrics** |
| --- | --- | --- | --- |
| Implementation Climate  *Evidence-Based Organizational Checklist* | - Measures the degree to which organizations expect, support, and reward EBT. Individual participants’ average scale scores are aggregated for all participants within a single organization to create an average score for the organization. - Supervisors and clinicians completed at baseline - Example items: “Executive leadership (e.g., administrators, directors) explicitly and repeatedly express support for and promote use of EBT”; “Clinicians are provided with EBT training opportunities and ready access to EBT materials (manuals, handouts, equipment).” | - 7 items - Scale:   1 = Never;  2 = Occasionally;  3 = Most of the time;  4=Ongoing/ Routine | - Internal reliability Cronbach’s   α = 0.86   - Construct validity ICC(1,1) = 0.41 |
| Participant Characteristics | - Collected information on a variety of demographic and descriptive information including age, sex, ethnicity, race, number of years they had conducted therapy, and whether they felt they mainly used EBTs in their work. - Participants indicated the total number of different types of training experiences they had with TF-CBT out of 12 possible options (e.g., “completed a 2-day in-person training,” “read the 2006 TF-CBT book”); experiences were summed. - Participants endorsed their primary theoretical orientation from a list of 10 possible options. - Supervisors provided an estimate of the percentage of time they spent providing supervision, whether they still actively performed clinical work, and chose the TF-CBT element that they felt was most difficult to supervise, which we transformed into a variable indicating whether or not they chose exposure as the most difficult element to supervise. |  |  |
| TF-CBT Self-efficacy  *TF-CBT Perceived Ability Scale* | - Measured self-reported ability to conduct TF-CBT - Supervisors and clinicians completed at baseline - Example items: “Please rate how competent you feel as a clinician… “Completing trauma narratives with children”, and “Analyzing complex clinical situations from a TF-CBT perspective.” | - 11 items - Scale:   1 = not at all;  2 = a little bit;  3 = somewhat;  4 = very much;  5 = exceptionally | - Internal reliability: Cronbach’s   α = .92   - Exploratory factor analysis using maximum likelihood extraction in the current sample justified retaining a single factor accounting for 56% of the variance |
| Declarative knowledge and skill with TF-CBT and exposure  *Skill in Implementing Components: Trauma and PTSD Scale* | - Measured declarative knowledge and skill with TF-CBT, adapted from two previous measures (Child Abuse Research Education and Service Institute, Rowan University, 2013, unpublished measure; National Crime Victims Research and Treatment Center, MUSC, 2010, unpublished measure). - Supervisors and clinicians completed at baseline - Example items: “Please choose the response that best describes your understanding and skill in implementing… “Psychoeducation (e.g., directive education about normal reactions to trauma)”; “In Vivo Exposure (desensitization to resolve avoidant behaviors/triggers in the environment)” | - 11 items - Scale:   0 = N/A (I do not use);  1 = Minimal;  2 = Minimal to moderate;  3 = Moderate;  4 = Moderate to advanced;  5 = Advanced | - Internal reliability: Cronbach’s   α = .91   - Exploratory factor analysis from earlier version of scale identified the 11 items retained for the current scale as one factor |
| TF-CBT Knowledge  *STEPS TF-CBT Knowledge Scale* | - Measures knowledge of TF-CBT using a multiple choice and true/false test questions combining items from the Denver Post Health Survey (Fitzgerald, 2010, unpublished measure, 2010) with items added by our team, and including content similar to the knowledge test used for the clinician TF-CBT certification program (<https://tfcbt.org>). - Supervisors and clinicians completed at baseline - Example items: “When teaching cognitive coping, wait to challenge distorted/unhelpful cognitions related to trauma.”; “In in vivo exposure, clinicians should do all of the following except…” | - 13 items - Scale: 8 questions are true/false, 5 questions are multiple choice | - Average item difficulty was 0.70 (range 0.31–0.93), meaning an average of 70% of respondents answered correctly. - Average item discrimination was 0.28 (range 0.14–0.37), demonstrating a good relation between the probability of answering each item correctly and the respondents’ total score (Dorsey, Pullmann, et al., 2017). - Convergent validity: TF-CBT knowledge was positively associated with the extensiveness of TF-CBT training reported by clinicians (r = .42, p <. 001) and the related, but distinct construct of TF-CBT efficacy (r = .27, p <. 001; (Dorsey, Pullmann, et al., 2017). |
| EBT Attitudes  *Modified Practice Attitude Scale (MPAS)* | - Measured attitudes toward EBTs (Borntrager, Chorpita, Higa-McMillan, & Weisz, 2009). - Supervisors and clinicians completed at baseline - Example items: “Clinical experience and judgment are more important than using evidence-based treatments,”; “Evidence-based treatments do not allow me to tailor my therapy to each client (reverse scored)” | - 5 items - Scale:   1 = Not at all;  2 = To a slight extent;  3 = To a moderate extent;  4 = To a great extent;  5 = To a very great extent | - Internal consistency Cronbach’s   α = 0.78   - Convergent validity with another measure of attitudes towards EBTs is moderate (*r* = .36; Borntrager, Chorpita, Higa-McMillan, & Weisz, 2009) |
| Supervision session time | - Length of time of supervision sessions recordings - Average minutes per case was calculated by dividing the total length of recording time by the number of cases discussed |  |  |
| Supervision content  *Supervision Process Observational Coding System (SPOCS)* | - Measured the intensity of supervision across multiple supervision content areas. Adapted from the Therapeutic Process Observational Coding System for Child Psychotherapy—Strategies scale (TPOCS-S; McLeod, 2001; McLeod & Weisz, 2010). The TPOCS-S categorizes psychotherapy treatment intervention elements using direct observation. Similarly, the SPOCS categorizes supervision elements, applying Garland et al.’s (2010) adaptation of the TPOCS-S by stratifying codes into content and technique domains. For the current study, we focused only on the content domain. - Supervision content was measured by 16 content areas: six practice elements common in many CBT-based interventions (assessment, psychoeducation, coping skills, exposure, cognitive processing, and behavior management); four items specific to TF-CBT and/or trauma treatment (e.g., trauma history, trauma-related safety); three general EBT techniques infrequently included in usual care (client homework, client role-play, and clinician modeling in session); two items frequently mentioned challenges in community settings (engagement and parent-level challenges); and one item to capture case management/other topics. - Three content areas were used in the current study: exposure, assessment, and “other topics” that were not TF-CBT specific, such as crisis/case management and small talk. - Exposure: the therapist and/or supervisor discuss and plan for TN, In Vivo, and/or gradual exposure with the child (e.g., integrating trauma-focused content into PRAC). Discussion may include reviewing how to deliver these components (e.g., identifying trauma triggers; engaging ways to do the TN; how to help a caregiver support a child in using relaxation when triggered), how the child responded to these components when delivered, and more general planning issues related to these components (e.g., documentation of the TN, what to do with it after treatment; how to incorporate GE into PRAC). - Low intensity example: “You should start the trauma narrative.” - High intensity would involve planning for the trauma narrative, how to avoid obstacles, practicing what to say, discussing how to involve the parent in the trauma narrative. - Assessment: the supervisor and/or clinician discuss information about the child’s psychiatric symptoms or behavior problems from standardized, formal assessment measures, or functional analysis (FA). This may include plans to administer standardized measures, clear planning to do FA, or feedback given to the client/caregiver from assessment or FA results and client/caregiver response. When coding assessment because of FA, the coder has to be sure that the clinician did, or will do FA (cannot make assumptions). Assessment also includes discussing assessment results and/or their meaning, and how results might inform treatment direction or speak to client progress through treatment (scores now compared to scores at the beginning of treatment/earlier in treatment). Assessment may also include measures assessing the caregiver’s functioning (depression, PTSD). - Other topics: This content item focuses on supervisor/therapist discussions that do not reflect the other TF-CBT content items. May include discussions of issues unrelated to the child’s traumatic experiences and/or not directly related to TF-CBT components. In order to rate this item as occurring, there needs to be a non-TF-CBT focused discussion between the supervisor/therapist. Although the range of topics covered under this topic vary from handling child suicidal ideation and case management to other, more “off-topic” discussion, this content item attempts to capture any content that is not one of the TF-CBT components or therapist technique content items. | - Trained coders rated occurrence of content areas in 5-minute intervals (low, medium, or high), and used these to determine intensity scores that provide overall dosage information about each content area (McLeod & Weisz, 2010). - Scale:   0 = not at all present;  1-2 = low intensity/brief mention;  3-4 = medium intensity/ acceptable coverage;  5-6 = high intensity/ thoroughly covered | Interrater reliability ICC_(2,6)_   - Overall = .87 - Exposure = .92 - Assessment = .76 - Other topics = .85 |
